# Exploring the Association between Vascular Dysfunction and Skeletal Muscle Mass, Strength and Function in Healthy Adults: A Systematic Review

**DOI:** 10.3390/nu12030715

**Published:** 2020-03-07

**Authors:** Svyatoslav Dvoretskiy, Jacqueline C. Lieblein-Boff, Satya Jonnalagadda, Philip J. Atherton, Bethan E. Phillips, Suzette L. Pereira

**Affiliations:** 1 Department of Kinesiology and Community Health, University of Illinois, Urbana-Champaign, IL 61801, USA; svyatoslav.dvoretskiy@abbott.com; 2 Abbott Nutrition, Columbus, OH 43219, USA; jacqueline.boff@abbott.com (J.C.L.-B.); satya.jonnalagadda@abbott.com (S.J.); 3 MRC-ARUK Centre of Excellence for Musculoskeletal Ageing Research, School of Medicine, University of Nottingham, Derby DE22 3DT, UK; Philip.Atherton@nottingham.ac.uk (P.J.A.); Beth.Phillips@nottingham.ac.uk (B.E.P.)

**Keywords:** vascular dysfunction, skeletal muscle mass, healthy adults

## Abstract

*Background*: The prevalence of vascular dysfunction increases with advancing age, as does the loss of muscle mass, strength and function. This systematic review explores the association between vascular dysfunction and skeletal muscle health in healthy adults. *Methods*: EMBASE and MEDLINE were searched for cross-sectional and randomized controlled studies between January 2009 and April 2019, with 33 out of 1246 studies included based on predefined criteria. Assessments of muscular health included muscle mass, strength and function. Macrovascular function assessment included arterial stiffness (pulse wave velocity or augmentation index), carotid intima-media thickness, and flow-mediated dilation. Microvascular health assessment included capillary density or microvascular flow (contrast enhanced ultrasound). *Results*: All 33 studies demonstrated a significant association between vascular function and skeletal muscle health. Significant negative associations were reported between vascular dysfunction and -muscle strength (10 studies); -mass (9 studies); and -function (5 studies). Nine studies reported positive correlations between muscle mass and microvascular health. *Conclusions*: Multiple studies have revealed an association between vascular status and skeletal muscle health in healthy adults. This review points to the importance of screening for muscle health in adults with vascular dysfunction with a view to initiating early nutrition and exercise interventions to ameliorate functional decline over time.

## 1. Introduction

The aging process is responsible for a variety of detrimental physiological changes within the human body, including losses of skeletal muscle mass, strength and function (termed sarcopenia) [1,2]. Over time, there is a progressive decline in the number and size of muscle fibers resulting in a total decrease in muscle mass of around 40% between the ages of 25 and 80 years [3]. The numerous negative consequences of sarcopenia are well-established and include an increased risk of falls, fractures, hospitalization, frailty, decreased quality of life and even death [4,5]. As such, sarcopenia has been shown to pose a significant economic burden to health care systems [6]. The prevalence of sarcopenia in community dwelling older adults is estimated to range from 7 to 73.3% in long-term care homes and between 22% and 87% in assisted-living facilities [7,8]. In addition, the progression of sarcopenia is complex and multifactorial, with environmental influences such as physical inactivity, nutrient deficiencies and oxidative stress all postulated to have a role [1,9].

Aging is also a major risk factor for cardiovascular (CV) disease, accounting for 17.3 million deaths per year and projected to be 23.6 million by 2030 globally [10,11]. Multiple studies show a relationship between low muscle mass and increased risk of CV-related mortality [12,13,14]. CV disease includes diseases associated with both the heart and the vasculature, with vascular disease encompassing both macro- and microvascular dysfunction.

Even in disease-free aging, it is postulated that age-related declines in macrovascular blood flow to appendicular regions could play a substantial role in determining muscle health. Macrovascular flow is generally assessed via conduit artery function by measuring arterial blood flow via Doppler ultrasound, arterial stiffness (measured by pulse wave velocity (PWV) or augmentation index (AI)), carotid intima-media thickness, and/or flow-mediated dilation (FMD), which is an estimate of endothelial function. It has been demonstrated that older individuals exhibit 20–30% reductions in limb conduit artery blood flow under both post-absorptive [15] and postprandial conditions [16] when compared to younger adults, possibly due to endothelial dysfunction [17,18]. Such blunted blood flow responses may contribute to age-related declines in anabolic responses to feeding by reducing the delivery and/or utility of insulin and amino acids (AA) in muscle [19,20]. In addition, more recently, Rodriguez and colleagues examined pulse wave velocity as an indicator of arterial stiffness in a meta-analysis and demonstrated that lower muscle mass is significantly associated with increased arterial stiffness [21].

In addition to macrovascular blood flow, ‘microvascular/capillary’ blood flow or perfusion is a critical mediator of insulin and AA delivery to the muscle [22,23,24]. Microvascular blood flow is not routinely measured in clinical practice, but for research purposes can be assessed using contrast enhanced ultrasound (CEUS) [25]. Capillary density and endothelial function are also good indicators of microvascular function—both have been shown to decline with age and improve in response to exercise training [26,27]. It has been suggested that reduced microvascular blood flow may contribute to the anabolic resistance of muscle [28] which is observed with advancing age [20,29,30], however, data on this is not yet conclusive [31].

This systematic review compiles studies that examine both skeletal muscle and vascular health in healthy adult populations. However, the prevalence of hypertension in otherwise healthy adults is estimated to be ~29% with progressive increases due to age [32]. Consequently, we expanded the search to include hypertensive adults without other health complications. While the included studies demonstrate various relationships between skeletal muscle health and vascular health, it remains unclear as to whether there is a causal relationship between the two. Given that there are some shared risk factors for declines in both systems, the relationship between them should be explored in a systematic way [21]. One previous systematic review attempted to do this but focused specifically on arterial stiffness assessed through PWV and did not explore other indices of macro or microvascular status [21]. Therefore, the aim of this systematic review is to determine if there is a relationship between skeletal muscle health and both macro- and microvascular function.

## 2. Materials and Methods

### 2.1. Literature Search

**Figure 1:**
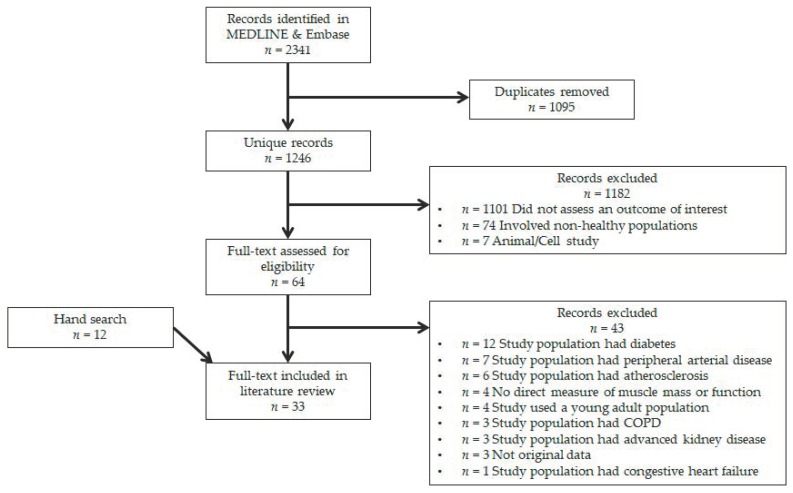
Study selection flow diagram.

The electronic databases EMBASE and MEDLINE were searched for original articles between January 2009 and April 2019. Evidence from cross-sectional and randomized controlled studies was included. All relevant articles were assessed for pre-defined criteria regarding study outcome, population and design. Database searching identified 2341 records of which 1095 were duplicates, leaving 1246 unique records for title and abstract screening. Following screening, 1182 records were excluded based on lack of assessment of an outcome of interest (*n* = 1101), inclusion of non-healthy participants (*n* = 74) and animal/cell studies (*n* = 7), leaving 64 records for full-text review. Of these, 43 records were excluded primarily due to inclusion of diseased participants (*n* = 32), leaving a total of 21 records. Through hand-searching full-text articles and references, we identified 12 more records which satisfied all criteria for inclusion. As such, a total of 33 records were included in this systematic review. A flow diagram is illustrated in Figure 1.

### 2.2. Inclusion Criteria

The primary inclusion criteria were based on the assessment of certain outcomes, specifically validated measurements of vascular function in combination with validated measurements of skeletal muscle health. Detailed descriptions of the included validated markers for skeletal muscle health assessments were split into three general categories: (1) muscle mass, (2) muscle strength and (3) muscle function and are described in Section 2.5. Arterial function measurements and markers that were included are described in Section 2.6.

Animal and cell-culture studies were not considered, nor were human studies involving diseased populations (diabetes, peripheral arterial disease, atherosclerosis, chronic obstructive pulmonary disease, kidney disease, cancer, or heart failure). Due to the prevalence of hypertension in otherwise healthy adults, the inclusion criteria did allow studies involving hypertensive adults with no other disease. Studies with professional athletic populations were not considered.

### 2.3. Data Extraction

Titles and abstracts resulting from the literature search were evaluated by two independent investigators. Disagreements were resolved by consensus, or by consulting with a third investigator if required. Studies were grouped according to skeletal muscle health assessments and vascular function assessments. Data were extracted and collated on the following study characteristics: reference, study design, participants (human, number of subjects, age, disease status, and gender ratio), skeletal muscle health measurements and vascular function measurements.

### 2.4. Study Details and Sample Demographics

All 33 studies were either cross-sectional or randomized controlled trials published within the past 10 years (Table 1). Twenty-three studies [20,26,27,33,34,35,36,37,38,39,40,41,42,43,44,45,46,47,48,49,50,51,52] included healthy mixed-age adults (age range 18–95) and two studies [53,54] included healthy community-dwelling older adults (age range 65–81). Six studies sampled participants with hypertension [55,56,57,58,59,60], one study sampled individuals with diabetes as a comparator to healthy aged population [61], and one study [62] did not report participant demographics. Study populations varied from a minimum of 6 [20] to a maximum of 3356 [42] participants. Eight studies included only men [25,27,37,42,49,50,56,61] and four studies included only women [38,52,53,60]. Most studies reported the age range of the participants, except for two studies with one claiming to study “middle-aged” adults [46] and one using adult participants [62].

### 2.5. Assessment of Skeletal Muscle Health

Muscle mass measurements were obtained by: (i) bioelectrical impedance analysis (BIA), (ii) computed tomography (CT), (iii) dual-energy X-ray absorptiometry (DXA) and (iv). muscle fiber cross sectional area analyses (commonly measured through muscle biopsy analysis). Muscle strength assessments were limited to: (i) hand-grip strength, (ii) torque measurements using an isokinetic dynamometer, and (iii) repetition-maximum exercises. Muscle function assessments were largely varied and included: (i) arm extensibility, (ii) 40-foot walking speed, (iii) sit and reach, (iv) sit-to-stand, (v) timed up and go (TUG), (vi) 12-min walk distance, (vii) perceived fatigue after a fast-pace 400-m walk, (viii) 10-m walking speed and ix. aerobic capacity. Table 2 indicates details of the assessment tools used to estimate skeletal muscle health. Most studies reported a measurement of skeletal muscle strength [26,27,34,35,36,37,39,41,49,53,55,57,59,60]. Thirteen studies assessed muscle mass [20,25,27,34,39,42,43,44,47,48,54,58,61] and six studies assessed muscle fiber cross-sectional area (CSA) [33,46,47,56,61,62]. Seven studies examined muscle function [35,38,40,49,50,51,52], one study assessed muscular power [41] and one study assessed the muscle anabolic response [20]. Body composition was measured as whole body lean mass [27,34,35,38,39,40,42,43,44,47,48,49,51,52,54,58] or as appendicular lean mass [20,25,26,33,36,37,41,46,50,53,55,56,57,59,60,61,62]. To assess skeletal muscle mass various modalities were utilized including BIA [42,43,44,54,58], DXA [25,27,34,39,47,48] and CT [27,43,46,47,61]. Muscle biopsies were obtained from the vastus lateralis muscle to analyze muscle fiber CSA [27,33,56,61,62]. Muscular strength and power were measured using various assessments including hand-grip strength [35,36,37,49,53,55,57,59], leg extension one-repetition maximum (1-RM) [27,39,41,49,52], peak leg torque [26] and leg extension eight-repetition maximum (8-RM) [34,60]. The muscle anabolic response was measured as muscle protein synthesis rate [20]. Muscle function was the most varied assessment and included measurements of sit and reach [49,51], sit-to-stand [38], TUG [35], 12-min walk distance [35], perceived fatigue after a fast-pace 400-m walk [40], 40-foot walking speed [38], 10-m walking speed [49], aerobic capacity [52] and arm extensibility [50] (Table 2).

### 2.6. Assessment of Vascular Health

Common validated markers of arterial function were: (i) pulse wave velocity, (ii) augmentation index, (iii) carotid intima-media thickness and (iv). flow-mediated dilation. Table 3 indicates details of the assessment tools used to measure vascular health. Macrovascular and/or microvascular health was assessed in the included studies, with the majority examining macrovascular blood flow. Macrovascular health was primarily assessed as arterial stiffness using multiple measurements of either pulse wave velocity (PWV) [36,39,40,42,43,46,48,49,51,52,54,58,60], radial augmentation index [41,44,57], carotid intima-media thickness (CIMT) [35,37,38,50,59] or flow-mediated dilation (FMD) [41]. CIMT was assessed using ultrasound [35,37,38,50,59] and radial augmentation index was measured using applanation tonometry [41,44,57]. For the studies assessing PWV, seven studies assessed brachial-ankle PWV [36,43,46,48,51,54,60], four studies assessed carotid-femoral PWV [39,40,49,52] and two studies assessed carotid-ankle PWV [42,58]. PWV was measured using a volume-plethysmographic apparatus [36,43,46,48,51,52,54,60], an oscillometric apparatus [42,49,58] and using applanation tonometry [39,40]. FMD was assessed using ultrasound [34,41] and applanation tonometry [53]. Microvascular health was assessed primarily through histology staining to determine capillary density in a vastus lateralis muscle biopsy [26,27,33,47,56,61,62] and through microvascular blood flow using contrast-enhanced ultrasound (CEUS) with Sonovue microbubbles [25]. One study assessed muscle perfusion using a muscle oxygenation apparatus [55].

## 3. Results

### 3.1. Macrovascular Studies

Macrovascular studies focus on dysfunction at the level of the large conduit arteries, which transport blood away from the heart. Most studies included in this review focused on macrovascular analyses with detailed descriptions of the included macrovascular studies presented in Table 4.

Body composition, specifically muscle mass, appears to have a strong association with arterial health. Given that PWV is the most commonly used assessment of arterial stiffness, six studies used PWV as a measurement of arterial health. These studies were conducted in middle-aged and older adults and revealed a negative correlation between PWV and muscle mass [42,43,46,48,54,58]. Using the radial augmentation index as a measurement of arterial stiffness, a single study found an inverse relationship between limb muscle mass and arterial function [44]. These correlations are indicative of greater arterial stiffness in individuals with lower muscle mass. These publications collectively suggest that skeletal muscle mass has an association with arterial health, specifically arterial wall elasticity.

In addition to skeletal muscle mass, skeletal muscle strength is an important indicator of overall muscle health. Hand-grip strength has been correlated with changes in muscle function and joint disability scores [63], disease activity states [64] and even all-cause mortality [65]. Multiple studies in this review reported that increased CIMT, a common measurement of arterial stiffness, is associated with impaired hand-grip strength [35,37,59]. Using other measurements of arterial stiffness, such as radial augmentation index [57] and pulse wave velocity [36], other studies show the same association between higher arterial stiffness and lower grip strength. One included study demonstrated that higher PWV is associated with lower absolute and relative muscle strength assessed using selected weight machines [39]. This observation is important as it directly demonstrates whole body strength loss and not solely hand-grip strength impairment. One included study demonstrated that decreased muscle strength is associated with endothelial dysfunction [53]. The authors of this study suggest that endothelial function plays an important role in overall muscle health. Additionally, increased arterial stiffness measured through radial augmentation index is associated with impaired leg power during leg press exercise [41]. These studies provide evidence for an association between skeletal muscle strength and arterial dysfunction.

Impaired skeletal muscle function also shows an association with macrovascular dysfunction. Various mobility tests are predictive of the onset of disability, hospitalization and mortality [66,67,68,69,70]. This association is important given that many included studies in this review demonstrate the correlation between arterial stiffness and poor performance on tests of muscle function. For example, increased PWV is associated with increased fatigue during a 400-m walk test [40] and increased CIMT is associated with poor performance on the 40-foot walking speed test [38], 12-min walk test [35] and the timed up and go test [35]. In addition, studies show impairments in muscle flexibility with arterial dysfunction. Increased arterial stiffness is associated with poor performance on multiple flexibility tests including sit and reach [51], and arm extensibility tests [50]. Furthermore, increased arterial stiffness is associated with poor performance on a common test of muscle function, the sit-to-stand test [38]. These studies suggest that the overall muscle function is impaired with arterial dysfunction.

Most included studies examined healthy adult populations. Nonetheless, the prevalence of hypertension in otherwise healthy adults is estimated to be ~29% [32] and increases as people age. Therefore, we examined relevant studies that included hypertensive participants [55,56,57,58,59,60]. All six studies involved middle-aged and older adults. Five of the six studies reported that arterial stiffness had a significant negative association with muscular strength [57,59,60] and muscle mass [58]. These studies suggest an additional burden of muscle dysfunction in hypertensive populations.

Exercise interventions that increase muscle mass, strength and function were also found to increase overall macrovascular health. One study included a group of middle-aged women that were subjected to 12 weeks of aerobic training [52]. Following the training program, participants experienced a concomitant decrease in arterial stiffness and increase in muscular function through increased strength on various leg exercises and increased aerobic capacity on the cycle ergometer [52]. One group combined both aerobic and resistance exercise into 2 times per week workouts lasting 10 weeks [49]. Upon completion of the study, participants experienced a decrease in arterial stiffness and an increase skeletal muscle strength through significant increases in leg press, chest press, shoulder press, leg curl and seated row exercises [49]. One study subjected young, middle-aged and older participants to progressive resistance exercise training three times a week for twenty weeks [34]. Upon completion of the training, researchers reported significant improvements in skeletal muscle mass and strength, as well as leg blood in response to feeding and exercise [34]. Lastly, a 12-week stair climbing exercise intervention in hypertensive older women increased skeletal muscle strength measured by 8-RM on a leg extension machine and decreased arterial stiffness [60]. This study is noteworthy as it demonstrates that it is possible to improve skeletal muscle health together with macrovascular health despite the presence of hypertension. Overall, these studies show the concomitant positive response of the skeletal muscle and arterial function to exercise, suggesting an intimate connection between the two.

### 3.2. Microvascular Studies

Arteries branch into arterioles which further branch into capillaries, which are primarily responsible for distributing blood carrying nutrients and oxygen to muscle tissues beds. Thus, it is critical to examine the relationship between the muscle microvasculature and skeletal muscle health. Both higher capillary density and/or greater microvascular flow within the muscle (muscle perfusion) offers potential for greater diffusion of substrates, oxygen, hormones, and nutrients, thereby enhancing skeletal muscle mass and function. Detailed descriptions of the included microvascular studies are presented in Table 5.

Capillary density, a common marker of microvascular health, appears to have an association with skeletal muscle mass regardless of age. Most studies examined this association through muscle biopsy analysis. These studies demonstrate that increased capillary density is correlated with increased muscle mass across age groups [33,47,62], as well as with increase muscle strength [26,27]. Correspondingly, decrease capillary density was observed in sarcopenic individuals along with concomitant decline in exercise capacity [47,61]. Microvascular flow and function were also found to decline in populations with functional impairments such as aging and chronic disease [43,61].

Two studies in this review included microvascular blood flow analysis in hypertensive participants. One study showed that hypertensive older adults have significantly lower capillary to fiber ratio and muscle mass compared to healthy older controls [56]. Furthermore, adults with hypertension have a reduced tissue oxygen saturation compared to healthy controls and require a two-fold increase in blood pressure to produce equal amount of muscle torque compared to controls [55]. These studies suggest a microvascular impairment in hypertensive populations.

Exercise interventions that increase muscle mass, strength and function were also found to increase microvascular flow and capillary density. In addition to the included cross-sectional studies, several randomized controlled trials that involved an exercise intervention were included. These studies are important as they demonstrate the intimate relationship between vascular health and skeletal muscle health. Two studies examined microvascular function via capillary density and reported significant improvements following both resistance [27] and aerobic [26] training. Following 24 weeks of aerobic exercise, older adults experienced a concomitant increase in skeletal muscle strength and capillary density [26]. Another study demonstrated that older adults following 12 weeks of resistance exercise training experienced an increase in skeletal muscle mass, strength and capillary density [27]. One study examined nutrient delivery to the muscle following a bout of aerobic exercise and reported significant improvements in muscle protein synthesis as well as microvascular blood flow [20]. These data suggest that various types of exercise can significantly impact both the skeletal muscle function and microvascular health of the participants.

## 4. Discussion

Sarcopenia, the progressive loss of muscle mass, muscle function and physical performance, was previously associated with cardiovascular disease [12,13,14]. Given that many of the risk factors for sarcopenia and cardiovascular disease are shared, it is not surprising that there is an association between measures of vascular dysfunction and muscle health. Existing reviews solely target arterial stiffness as an indicator of vascular dysfunction and its’ association with skeletal muscle health [21]. This systematic review takes a broader approach and examines other validated indices of vascular function including microvascular function. The focus of the review is on relatively healthy adults and we observed an inverse association between skeletal muscle health and vascular dysfunction. Our data indicate that the inverse relationship between vascular dysfunction and skeletal muscle is consistently observed in the hypertensive populations as well. Given the high prevalence of hypertension (63.1%) in adults over the age of 60 [32], these data suggest that it may be important to start to screen for and address muscle health issues in adults with hypertension. Not unexpectedly, exercise interventions demonstrated both macrovascular and microvascular health benefits, in addition to improving indices of skeletal muscle health, indicating the importance of habitual exercise for healthy aging.

Given that most of the included studies are cross-sectional, the mechanism of the association between vascular dysfunction and muscle strength and function is not clear. Rodriguez and colleagues completed an extensive review of the musculoskeletal system and vasculature and proposed a conceptual disease model [71]. On the microsystemic level, systemic inflammation, local inflammation, low calcium and low vitamin D intake, as well as impaired glucose metabolism promote cellular stress and damage. If the cellular stress and damage is chronic, it is manifested in damage to both the vascular system, impacting endothelial function and eventually microvascular health and the musculoskeletal system [71]. Arterial stiffness and perhaps microvascular dysfunction would occur in combination with sarcopenia on the macrosystemic level. It has been shown that muscle perfusion, an indicator of peripheral microvascular health, decline with age [25]. This decline can lead to a decrease in ‘nutritive’ flow to the muscle, impacting the availability of nutrients need for muscle function [22,23,24]. Reduced ‘nutritive’ blood flow may contribute to the anabolic resistance of muscle observed with ageing [29,30], eventually leading to loss of muscle mass, strength and function. What is not known is if the relationship between vascular dysfunction and muscle health is bi-directional and this needs to be further explored in prospective studies.

There are some important limitations of the included studies that must be considered. First, there is a heterogeneity in assessment of arterial health. For example, arterial dysfunction in the included studies is measured through pulse-wave velocity, CIMT, augmentation index and flow-mediated dilation. This variation makes it difficult to complete a meta-analysis, though we felt it was important to include these studies to demonstrate the relationship between arterial dysfunction and skeletal muscle health regardless of outcome measurement. Second, like the variation in arterial function assessments, there is a heterogeneity in assessment of skeletal muscle health and function. While muscle mass and strength assessments are relatively consistent, muscle function assessments varied greatly from 40-foot walking speed tests [38] to arm extensibility tests [50]. Lastly, the number of studies evaluating microvascular health is comparatively low and more research is needed in this field.

The exclusion criteria for each study varied which impacts several variables including nutrition status, daily physical activity and use of medications. Though a few of the studies excluded participants based on a low BMI [4,25,59] and low daily protein intake [26], the rest of the studies did not examine the nutrition status of the participants. Nutrition has been suggested to play an important role in arterial stiffness, specifically through the dietary intake of vitamin D and calcium. An observational study conducted on 131 participants suggested that insufficient intake of vitamin D is associated with increased arterial stiffness [72]. A large study of 12,097 men and women determined that higher calcium intake from food was associated with decreased risk for stroke and non-fatal cardiovascular disease [73]. Despite these data, a detailed meta-analysis of vitamin D supplementation and the impact on arterial stiffness determined that there is inconsistent evidence to suggest a connection between the two factors, which was attributed to large heterogeneity in study design [74]. Similarly, other studies demonstrated no relationship between calcium supplementation and markers of vascular disease [75,76]. Most studies included in this systematic review did not measure daily physical activity. Given the importance of physical activity for maintaining skeletal muscle mass and improving cardiovascular function [77,78], an accurate measurement of daily activity is important for a complete assessment of the participants. Lastly, the use of medications was poorly reported in most studies and this can impact a variety of factors related to vascular dysfunction including blood pressure, blood triglycerides and blood cholesterol levels.

Although the focus of this systematic review has been on relatively healthy subjects, the association between skeletal muscle health and vascular dysfunction has been demonstrated in other populations. A recent meta-analysis examined macrovascular function through pulse wave velocity and demonstrated that lower muscle tissue is associated with higher arterial stiffness in populations with diabetes and kidney disease [21]. This association has been demonstrated in healthy children as well, in which researchers determined that arterial stiffness (measured by CIMT) is negatively associated with muscular strength [79]. Furthermore, following isometric handgrip training, young healthy adults improved their brachial artery flow-mediated dilation [80]. Overall, the connection between skeletal muscle function and vascular dysfunction is observed in other populations and may be exacerbated in populations with disease.

## 5. Conclusions

In conclusion, we described over 30 studies that demonstrate an inverse relationship between vascular dysfunction and skeletal muscle health. This association is observed both on the macrovascular and the microvascular levels. Given the cross-sectional nature of most of the studies included, it is impossible to say if impaired vascular health causes skeletal muscle dysfunction or vice versa. Nevertheless, we included some exercise intervention trials that demonstrate concurrent improvements in skeletal muscle health and vascular function [26,27,49,52,60]. More studies are necessary to determine if vascular dysfunction is predictive of skeletal muscle dysfunction. Furthermore, the field requires a standardized assessment of macrovascular dysfunction and should also start to include assessment of microvascular dysfunction. The clinical significance of this association between vascular health and muscle health cannot be overlooked, considering the heavy clinical and economic burden of vascular dysfunction and sarcopenia, independently. If skeletal muscle health impairment is predictive of future cardiovascular events or vice versa, early screenings will allow for early preventative interventions to help improve long-term outcomes as the population ages. It is possible that interventions targeting vascular dysfunction may have a long-term benefit on muscle health or vice versa, but this needs to be systematically tested in prospective randomized clinical studies.

## Figures and Tables

**Table 1:** Study details and sample demographics.

Study & Year	Design	Sample	Country	*n*	Male (%)	Age	Exclusion
Barnouin 2017 [33]	Cross-sectional	Healthy adults	UK	47	77	22–74	Cardiovascular, neuromuscular, or respiratory diseases
Barrera 2014 [35]	Cross-sectional	Healthy adults	Chile	259	49	29–88	Undernutrition BMI < 18, cancer, autoimmune disease, kidney, liver or cardiac failure, diabetes, cognitive impairment, steroids or HRT
Brightwell 2018 [26]	RCT	Healthy adults	USA	23	30	65–82	Diabetes, cancer, smoking, CVD, kidney disease, uncontrolled high blood pressure, low daily protein intake
Chung 2018 [36]	Cross-sectional	Healthy adults	Korea	1590	78	40–79	Metabolic syndrome, HRT, any medication
den Ouden 2013 [37]	Cross-sectional	Healthy adults	The Netherlands	403	100	73–91	Inability to visit the study center independently
Dipla 2017 [55]	Cross-sectional	Healthy and hypertensive adults	Greece	91	60	31–55	CVD, diabetes
Fahs 2017 [39]	Cross-sectional	Healthy adults	USA	71	51	18–75	Hypertension, participation in regular exercise, HRT
Gonzales 2015 [40]	Cross-sectional	Healthy adults	USA	45	44	60–78	CVD, diabetes, pulmonary disease, HRT, obesity, medication for blood pressure or cholesterol
Groen 2014 [61]	Cross-sectional	Healthy and T2DM adults	The Netherlands	45	100	23–71	Impaired renal or liver function, obesity, CVD, hypertension, advanced diabetes, insulin therapy
Gueugneau 2016 [56]	Cross-sectional	Healthy and hypertensive adults	France	37	100	21–74	Prior myocardial infarction or stroke, heart failure, atrial fibrillation, diabetes, morbid obesity, Parkinson’s disease
Heffernan 2012 [41]	Cross-sectional	Healthy adults	USA	24	46	70–85	Acute/terminal illness, coronary heart disease, myocardial infarction, hypertension, neuromuscular disease, HRT, diabetes, renal disease, BMI > 32.5
Im 2017 [42]	Cross-sectional	Healthy adults	Korea	3356	100	40–64	ABI < 0.9, high WBC count, cancer
Khoudary 2015 [38]	Cross-sectional	Healthy adults	USA	1103	0	56–62	Stroke, angina or myocardial infarction, hysterectomy or bilateral oophorectomy, pregnancy, HRT
Kohara 2017 [43]	Cross-sectional	Healthy adults	Japan	1518	40	60–74	CVD, PAD, stroke, coronary heart disease, and congestive heart failure
Lee 2014 [44]	Cross-sectional	Healthy adults	Korea	427	42	52–95	n/r or unclear
Lima-Junior 2018 [57]	Cross-sectional	Hypertensive adults	Brazil	72	28	48–68	<18 years, smoking, diabetics, CVD, inability to perform isometric handgrip, enrolled in physical activity program
Mitchell 2013 [25]	RCT	Healthy adults	UK	36	100	18–75	Diabetes, CVD, BMI < 18 or >28
Ochi 2010 [46]	Cross-sectional	Healthy adults	Japan	496	36	n/r	Stroke, TIA, coronary heart disease and congestive heart failure
Phillips 2012 [34]	RCT	Healthy adults	UK	51	57	21–72	Muscle wasting, metabolic or respiratory diseases, CVD, chronic diseases
Prior 2016 [47]	Cross-sectional	Healthy adults	USA	76	42	45–80	Coronary artery disease, heart failure, PAD, stroke, liver, kidney or lung disease, smoking
Sampaio 2014 [58]	Cross-sectional	Healthy and hypertensive adults	Japan	175	48	70–77	Moderate cognitive impairment, uncontrolled cardiovascular, pulmonary or metabolic diseases, stroke, Parkinson’s disease, PAD, orthopedic disease
Sanada 2010 [48]	Cross-sectional	Healthy adults	Japan	1488	29	18–85	CVD, beta-blockers, HRT, athletes
Shimizu 2017 [59]	Cross-sectional	Hypertensive adults	Japan	795	57	60–89	Participants without hypertension, BMI < 18.5, high BP, history of stroke
Shiotsu 2018 [49]	RCT	Healthy adults	Japan	45	100	63–85	Current participation in structured exercise program, CVD, musculoskeletal disease, diabetes
Suwa 2018 [50]	Cross-sectional	Healthy adults	Japan	1354	100	35–59	CVD, history of stroke
Timmerman 2012 [20]	RCT	Healthy older adults	USA	6	50	67–73	Obesity, chronic diseases
Verdijk 2016 [27]	RCT	Healthy adults	The Netherlands	30	100	19–83	CVD, PAD, diabetes, inability to participate in an exercise program
Wong 2018 [60]	RCT	Hypertensive adults	Korea	41	0	49–67	Pre-menopause, CVD, diabetes, HRT, smoking, exercise, endocrine disorders, psychiatric disorders
Wüst 2009 [62]	Cross-sectional	Adults	UK	11	45	n/r	n/r or unclear
Yamamoto 2009 [51]	Cross-sectional	Healthy adults	Japan	526	34	20–83	Obesity, chronic diseases, smoking, ABI < 0.9, any medication
Yoo 2018 [53]	Cross-sectional	Community-dwelling older adults	Korea	236	0	67–79	CVD, cognitive disorder, malignancy
Yoshizawa 2009 [52]	RCT	Healthy adults	Japan	35	0	32–59	Chronic diseases, smoking, any medication
Zhang 2019 [54]	Cross-sectional	Community-dwelling older adults	China	1002	42	65–81	n/r or unclear

Abbreviations: BMI: Body Mass Index; RCT: Randomized Controlled Trial; HRT: Hormone Replacement Therapy; CVD: Cardiovascular Disease; T2DM: Type 2 Diabetes Mellitus; ABI: Ankle-Brachial Index; WBC: White Blood Cells; PAD: Peripheral Artery Disease; TIA: Transient Ischemic Attack; n/r: Not reported.

**Table 2:** Assessment of skeletal muscle health.

Study & Year	Parameter	Region	Modality	Device
Brightwell 2018 * [26]	Muscular strength	Appendicular	Peak leg torque	Biodex isokinetic dynamometer
Chung 2018 [36]	Muscular strength	Appendicular	Hand-grip strength	Hand-grip dynamometer
den Ouden 2013 [37]	Muscular strength	Appendicular	Hand-grip strength	JAMAR hand-grip dynamometer
Dipla 2017 [55]	Muscular strength	Appendicular	Hand-grip strength	Biopac hand-grip dynamometer
Lima-Junior 2018 [57]	Muscular strength	Appendicular	Hand-grip strength	Hand-grip dynamometer
Shimizu 2017 [59]	Muscular strength	Appendicular	Hand-grip strength	Smedley hand-grip dynamometer
Wong 2018 [60]	Muscular Strength	Appendicular	8-RM	Cybex dynamometer
Yoo 2018 [53]	Muscular strength	Appendicular	Hand-grip strength	T.K.K hand-grip dynamometer
Im 2017 [42]	Muscle mass	Whole body	BIA	Inbody
Kohara 2017 [43]	Muscle mass	Whole body	BIA, CT	Omron, GE
Lee 2014 [44]	Muscle mass	Whole body	BIA	InBody
Mitchell 2013 * [25]	Muscle mass	Appendicular	DXA	Lunar
Sampaio 2014 [58]	Muscle mass	Whole body	BIA	n/r
Sanada 2010 [48]	Muscle mass	Whole body	DXA	Hologic
Timmerman 2012 * [20]	Muscle anabolic response	Appendicular	Muscle biopsy	Bergström needle
Zhang 2019 [54]	Muscle mass	Whole body	BIA	InBody
Barnouin 2017 * [33]	Muscle CSA	Appendicular	Muscle biopsy	Bergström needle
Gueugneau 2016 * [56]	Muscle CSA	Appendicular	Muscle biopsy	Bergström needle
Ochi 2010 [46]	Muscle CSA	Appendicular	CT	GE
Wüst 2009 * [62]	Muscle CSA	Appendicular	Muscle biopsy	Percutaneous needle
Gonzales 2015 [40]	Muscular function	Whole body	400 m walk	Polar monitor (to track HR during walk)
Khoudary 2015 [38]	Muscular function	Whole body	40-foot walking speed, sit-to-stand test	n/r
Suwa 2018 [50]	Muscular function	Appendicular	Arm extensibility test	n/r
Yamamoto 2009 [51]	Muscular function	Whole body	Sit and reach test	Takei Scientific digital flexibility testing device
Yoshizawa 2009 [52]	Muscular function	Whole body	1-RM, aerobic capacity	Selectorized weight machines, cycle ergometer
Barrera 2014 [35]	Muscular strength and function	Whole body	12-min walk, TUG, hand-grip strength	Digital force transducer and hand-grip dynamometer
Fahs 2017 [39]	Muscular strength and muscle mass	Whole body	1-RM, DXA	Selectorized weight machines, Hologic
Groen 2014 * [61]	Muscle mass and CSA	Appendicular	CT, muscle biopsy	n/r, percutaneous needle
Heffernan 2012 [41]	Muscle strength and power	Appendicular	1-RM	Keiser Sports
Phillips 2012 [34]	Muscle mass and strength	Whole body	DXA, leg extension 75% 1-RM	Lunar, Leisure Lines
Prior 2016 * [47]	Muscle mass and CSA	Whole body	DXA, CT	Lunar, Siemens
Shiotsu 2018 [49]	Muscular strength and function	Whole body	1-RM, hand-grip strength, 10-m walk, sit and reach test	Leg press/curl, chest/shoulder press, seated row, hand-grip dynamometer
Verdijk 2016 * [27]	Muscular strength and muscle mass	Whole body	1-RM, CT, DXA, muscle biopsy	Technogym, Phillips Medical, GE, percutaneous needle

* Microvascular only assessment (no asterisk indicates macrovascular only); Abbreviations: RM: Repetition Maximum; BIA: Bioelectrical Impedance Analysis; CT: Computed Tomography; DXA: Dual-Energy X-ray Absorptiometry; CSA: Muscle Fiber Cross-Sectional Area; TUG: The Timed Up and Go Test; n/r: Not reported.

**Table 3:** Assessment of vascular health.

Study & Year	Parameter	Vascular Site	Method	Device
Chung 2018 [36]	PWV	Brachial-ankle	Volume-plethysmographic apparatus	Colin Medical
Fahs 2017 [39]	PWV	Carotid-femoral	Applanation tonometry	SphygmoCor
Gonzales 2015 [40]	PWV	Carotid-femoral	Applanation tonometry	SphygmoCor
Im 2017 [42]	PWV	Carotid-ankle	Oscillometric	Fukuda Denshi
Kohara 2017 [43]	PWV	Brachial-ankle	Volume-plethysmographic apparatus	Omron
Ochi 2010 [46]	PWV	Brachial-ankle	Volume-plethysmographic apparatus	Omron
Sampaio 2014 [58]	PWV	Carotid-ankle	Oscillometric	Fukuda Denshi
Sanada 2010 [48]	PWV	Brachial-ankle	Volume-plethysmographic apparatus	Colin Medical
Shiotsu 2018 [49]	PWV	Carotid-femoral	Oscillometric	Fukuda Denshi
Wong 2018 [60]	PWV	Brachial-ankle	Volume-plethysmographic apparatus	Colin Medical
Yamamoto 2009 [51]	PWV	Brachial-ankle	Volume-plethysmographic apparatus	Omron
Yoshizawa 2009 [52]	PWV	Carotid-femoral	Volume-plethysmographic apparatus	Colin Medical
Zhang 2019 [54]	PWV	Brachial-ankle	Volume-plethysmographic apparatus	Omron
Barrera 2014 [35]	CIMT	Carotid	Ultrasound	GE
den Ouden 2013 [37]	CIMT	Carotid	Ultrasound	ATL Ultramark IV
Khoudary 2015 [38]	CIMT	Carotid	Ultrasound	Teratech Corp
Shimizu 2017 [59]	CIMT	Carotid	Ultrasound	GE
Suwa 2018 [50]	CIMT	Carotid	Ultrasound	Aplio
Heffernan 2012 [41]	Aix	Radial	Applanation tonometry	Omron
Lee 2014 [44]	Aix	Radial	Applanation tonometry	SphygmoCor
Lima-Junior 2018 [57]	Aix	Radial	Applanation tonometry	EndoPAT
Dipla 2017 [55]	FMD	Brachial	Muscle oxygenation apparatus	NIRS Artinis
Yoo 2018 [53]	FMD	Brachial	Applanation tonometry	EndoPAT
Phillips 2012 [34]	LBF	Femoral	Doppler ultrasound	Toshiba
Barnouin 2017 * [33]	C: F Ratio	Femoral	Immunohistochemistry	Bergström needle
Brightwell 2018 * [26]	C: F Ratio	Femoral	Immunohistochemistry	Bergström needle
Groen 2014 * [61]	C: F Ratio	Femoral	Immunohistochemistry	Percutaneous needle
Gueugneau 2016 * [56]	C: F Ratio	Femoral	Immunohistochemistry	Bergström needle
Prior 2016 * [47]	C: F Ratio	Femoral	Immunohistochemistry	Percutaneous needle
Verdijk 2016 * [27]	C: F Ratio	Femoral	Immunohistochemistry	Percutaneous needle
Wüst 2009 * [62]	C: F Ratio	Femoral	Immunohistochemistry	Percutaneous needle
Mitchell 2013 * [25]	MBF	Femoral	Contrast-enhanced ultrasound	Sonovue
Timmerman 2012 * [20]	MBF	Femoral	Doppler ultrasound	Philips ATL

* Microvascular only assessment (no asterisk indicates macrovascular only); Abbreviations: PWV: Pulse Wave Velocity; CIMT: Carotid Intima Media Thickness; Aix: Radial Augmentation Index; FMD: Flow Mediated Dilation; LBF: Leg Blood Flow; C: F Ratio: Capillary to Fiber Ratio; MBF: Microvascular Blood Flow.

**Table 4:** Association between skeletal muscle and macrovascular blood flow.

Study & Year	Sample	Age	Muscle and Vascular Association	Type of Association	Finding
Barrera 2014 [35]	Healthy adults	29–88	Muscular strength/function and CIMT	Difference between groups (*p* < 0.05) ^†^	In older adults, CIMT is negatively associated with muscular strength and function
Chung 2018 [36]	Healthy adult men	40–79	Muscular strength and PWV	Difference between groups (*p* < 0.05) ^†^	In middle-aged and older adults, arterial stiffness is negatively associated with muscular strength and function
den Ouden 2013 [37]	Healthy older men	73–91	Muscular strength and CIMT	Correlation (r = −0.17; *p* < 0.05)	In older men, CIMT is negatively associated with muscular strength
Dipla 2017 ^#^ [55]	Healthy and hypertensive adults	31–55	Muscular strength and muscle perfusion	Difference between groups (*p* < 0.05) ^†^	Hypertensive adults have reduced tissue oxygen saturation compared to healthy controls; to produce same amount of torque compared to healthy controls requires a two-fold increase in BP
Fahs 2017 [39]	Healthy adults	18–75	Muscular strength and PWV	Correlation (*r* = −0.230/−0.484; *p* < 0.05)	In adults, arterial stiffness is negatively correlated with absolute and relative muscular strength
Gonzales 2015 [40]	Healthy older adults	60–78	Muscular function and PWV	Beta coefficient (*p* < 0.05)	In older adults, arterial stiffness is positively correlated with muscle fatigue
Heffernan 2012 [41]	Healthy older adults	70–85	Muscular power and augmentation index	Correlation (*r* = -0.54; *p* < 0.05)	In older adults, arterial stiffness is negatively associated with muscular power
Im 2017 [42]	Healthy adult men	40–64	Muscle mass and PWV	Correlation (*p* < 0.05)	In middle-aged men, arterial stiffness is negatively correlated with muscle mass
Khoudary 2015 [38]	Healthy older women	56–62	Muscle function and CIMT	Beta coefficient (0.028; *p* < 0.05)	In older women, CIMT is negatively associated with muscle function
Kohara 2017 [43]	Healthy older adults	60–74	Muscle mass and PWV	Correlation (r = −0.24; *p* < 0.05)	In older adults, arterial stiffness is negatively correlated with muscle mass
Lee 2014 [44]	Healthy older adults	52–95	Muscle mass and augmentation index	Beta coefficient (*p* < 0.05)	In older adults, arterial stiffness is negatively associated with muscle mass
Lima-Junior 2018 ^#^ [57]	Hypertensive older adults	48–68	Muscular strength and augmentation index	Beta coefficient (−0.49; *p* < 0.05)	In older adults with hypertension, arterial stiffness is negatively associated with muscular strength
Ochi 2010 [46]	Healthy adults	n/r	Muscle CSA and PWV	Correlation (*r* = −0.34; *p* < 0.05)	In men, arterial stiffness is negatively associated with muscle mass
Phillips 2012 * [34]	Healthy adults—resistance exercise	21–72	Muscle mass/strength and leg blood flow	Difference between groups (*p* < 0.05) ^†^	Following resistance exercise training, adults experience increases in leg blood flow, muscle mass and strength regardless of age in response to feeding
Sampaio 2014 ^#^ [58]	Healthy and hypertensive older adults	70–77	Muscle mass and PWV	Odds ratio (1.82; *p* < 0.05)	In healthy and hypertensive older adults, arterial stiffness is negatively associated with muscle mass
Sanada 2010 [48]	Healthy adults	41–71	Muscle mass and PWV	Difference between groups (*p* < 0.05) ^†^	Women with sarcopenia have higher arterial stiffness compared to healthy controls
Shimizu 2017 ^#^ [59]	Hypertensive older adults	60–89	Muscular strength and CIMT	Difference between groups (*p* < 0.05) ^†^	In older adults with hypertension, CIMT is negatively associated with muscular strength
Shiotsu 2018 * [49]	Healthy older men—resistance exercise	63–85	Muscular strength/function and PWV	Difference between groups (*p* < 0.05) ^†^	Following resistance exercise training, older men experience a decrease in arterial stiffness and an increase in muscular strength/function
Suwa 2018 [50]	Healthy adult men	35–59	Muscular function and CIMT	Beta coefficient (−0.189; *p* < 0.05)	In middle-aged adults, CIMT is negatively associated with arm flexibility
Wong 2018 *^#^ [60]	Hypertensive older women—stair climbing exercise	49–67	Muscular strength and PWV	Correlation (r = −0.47; *p* < 0.05)	Following stair climbing training, hypertensive older women experience a decrease in arterial stiffness and an increase in muscular strength
Yamamoto 2009 [51]	Healthy adults	40–83	Muscular function and PWV	Correlation (r = 0.17/0.45; *p* < 0.05)	In middle-aged and older adults, arterial stiffness is negatively correlated with flexibility
Yoo 2018 [53]	Older women	67–79	Muscular strength and endothelial function	Correlation (r = 0.176; *p* < 0.05)	After adjusting for comorbidities, in older women, endothelial function is positively correlated with muscular strength
Yoshizawa 2009 * [52]	Healthy women—aerobic exercise	32–59	Muscular function and PWV	Difference between groups (*p* < 0.05) ^†^	Following aerobic training, middle-aged women experience a decrease in arterial stiffness and an increase in muscular function
Zhang 2019 [54]	Older adults	65–81	Muscle mass and PWV	Odds ratio (1.11; *p* < 0.05)	After adjusting for comorbidities, in older adults, arterial stiffness is negatively associated with muscle mass

* Exercise intervention study; ^#^ hypertensive population; ^†^ correlation coefficient not reported; Abbreviations: CIMT: Carotid Intima-Media Thickness; PWV: Pulse Wave Velocity; CSA: Mid-thigh Muscle Cross-sectional Area; n/r: Not reported.

**Table 5:** Association between skeletal muscle and microvascular blood flow.

Study & Year	Sample	Age	Muscle and Vascular Association	Type of Association	Finding
Barnouin 2017 [33]	Healthy adults	22–74	Muscle fiber CSA and capillary density	Correlation (R^2^ = 0.46; *p* < 0.05)	In young and older adults, capillary-to-fiber ratio is positively correlated with muscle mass
Brightwell 2018 * [26]	Healthy older adults—aerobic exercise	65–82	Muscular strength and capillary density	Difference between groups (*p* < 0.05) ^†^	Following aerobic training, older adults experience an increase in capillary density and an increase in muscular strength
Groen 2014 [61]	Healthy adult men	23–71	Muscle fiber CSA and capillary density	Difference between groups (*p* < 0.05) ^†^	Older adults have reduced capillary-to-fiber ratio and muscle mass compared to young controls
Gueugneau 2016 ^#^ [56]	Healthy and hypertensive older men	72–74	Muscle fiber CSA and capillary density	Difference between groups (*p* < 0.05) ^†^	Older adults with hypertension have lower capillary-to-fiber ratio and muscle mass compared to healthy older controls
Mitchell 2013 [25]	Healthy adult men	18–75	Muscle mass and microvascular blood flow	Difference between groups (*p* < 0.05) ^†^	Young adults have higher muscle mass and have higher microvascular blood flow in response to feeding compared to healthy older adults
Prior 2016 [47]	Healthy adults	45–80	Muscle mass and capillary density	Correlation (r = 0.30–0.37; *p* < 0.05)	In adults, capillary-to-fiber ratio is positively correlated with muscle mass
Timmerman 2012 [20]	Healthy older adults—aerobic exercise	67–73	Muscle protein synthesis and microvascular blood flow	Difference between groups (*p* < 0.05) ^†^	Following aerobic exercise, older adults experience improved microvascular flow and muscle protein synthesis
Verdijk 2016 * [27]	Healthy older adults—resistance exercise	65–83	Muscle fiber CSA/strength and capillary density	Difference between groups (*p* < 0.05) ^†^	Following resistance training, older adults experience an increase in capillary-to-fiber ratio and an increase in muscle mass and strength
Wüst 2009 [62]	Adults	n/r	Muscle fiber CSA and capillary density	Correlation (*r* = 0.62; *p* < 0.05)	In adults, capillary-to-fiber ratio is positively correlated with muscle mass

* Exercise intervention study; ^#^ hypertensive population; ^†^ correlation coefficient not reported; Abbreviations: CSA: Mid-Thigh Muscle Cross-sectional area.

## References

[1] Cruz-Jentoft A.J., Bahat G., Bauer J., Boirie Y., Bruyere O., Cederholm T., Cooper C., Landi F., Rolland Y., Sayer A.A. (2019). Sarcopenia: Revised European consensus on definition and diagnosis. Age Ageing.

[2] Studenski S.A., Peters K.W., Alley D.E., Cawthon P.M., McLean R.R., Harris T.B., Ferrucci L., Guralnik J.M., Fragala M.S., Kenny A.M. (2014). The FNIH sarcopenia project: Rationale, study description, conference recommendations, and final estimates. J. Gerontol. Ser. A Biol. Sci. Med Sci..

[3] Deschenes M.R. (2004). Effects of aging on muscle fibre type and size. Sports Med..

[4] Beaudart C., Rizzoli R., Bruyere O., Reginster J.Y., Biver E. (2014). Sarcopenia: Burden and challenges for public health. Arch. Public Health.

[5] Scott D., Daly R.M., Sanders K.M., Ebeling P.R. (2015). Fall and Fracture Risk in Sarcopenia and Dynapenia With and Without Obesity: The Role of Lifestyle Interventions. Curr. Osteoporos. Rep..

[6] Goates S., Du K., Arensberg M.B., Gaillard T., Guralnik J., Pereira S.L. (2019). Economic Impact of Hospitalizations in US Adults with Sarcopenia. J. Frailty Aging.

[7] Locquet M., Beaudart C., Petermans J., Reginster J.Y., Bruyere O. (2019). EWGSOP2 Versus EWGSOP1: Impact on the Prevalence of Sarcopenia and Its Major Health Consequences. J. Am. Med. Dir. Assoc..

[8] Rodriguez-Rejon A.I., Ruiz-Lopez M.D., Wanden-Berghe C., Artacho R. (2019). Prevalence and Diagnosis of Sarcopenia in Residential Facilities: A Systematic Review. Adv. Nutr..

[9] Dent E., Morley J.E., Cruz-Jentoft A.J., Arai H., Kritchevsky S.B., Guralnik J., Bauer J.M., Pahor M., Clark B.C., Cesari M. (2018). International Clinical Practice Guidelines for Sarcopenia (ICFSR): Screening, Diagnosis and Management. J. Nutr. Health Aging.

[10] Laslett L.J., Alagona P., Clark B.A., Drozda J.P., Saldivar F., Wilson S.R., Poe C., Hart M. (2012). The worldwide environment of cardiovascular disease: Prevalence, diagnosis, therapy, and policy issues: A report from the American College of Cardiology. J. Am. Coll. Cardiol..

[11] Smith S.C., Collins A., Ferrari R., Holmes D.R., Logstrup S., McGhie D.V., Ralston J., Sacco R.L., Stam H., Taubert K. (2012). Our time: A call to save preventable death from cardiovascular disease (heart disease and stroke). J. Am. Coll. Cardiol..

[12] Atkins J.L., Whincup P.H., Morris R.W., Lennon L.T., Papacosta O., Wannamethee S.G. (2014). Sarcopenic obesity and risk of cardiovascular disease and mortality: A population-based cohort study of older men. J. Am. Geriatr. Soc..

[13] Chuang S.Y., Chang H.Y., Lee M.S., Chia-Yu Chen R., Pan W.H. (2014). Skeletal muscle mass and risk of death in an elderly population. Nutr. Metab. Cardiovasc. Dis..

[14] Spahillari A., Mukamal K.J., DeFilippi C., Kizer J.R., Gottdiener J.S., Djousse L., Lyles M.F., Bartz T.M., Murthy V.L., Shah R.V. (2016). The association of lean and fat mass with all-cause mortality in older adults: The Cardiovascular Health Study. Nutr. Metab. Cardiovasc. Dis..

[15] Donato A.J., Uberoi A., Wray D.W., Nishiyama S., Lawrenson L., Richardson R.S. (2006). Differential effects of aging on limb blood flow in humans. Am. J. Physiol. Heart Circ. Physiol..

[16] Skilton M.R., Lai N.T., Griffiths K.A., Molyneaux L.M., Yue D.K., Sullivan D.R., Celermajer D.S. (2005). Meal-related increases in vascular reactivity are impaired in older and diabetic adults: Insights into roles of aging and insulin in vascular flow. Am. J. Physiol. Heart Circ. Physiol..

[17] Celermajer D.S., Sorensen K.E., Spiegelhalter D.J., Georgakopoulos D., Robinson J., Deanfield J.E. (1994). Aging is associated with endothelial dysfunction in healthy men years before the age-related decline in women. J. Am. Coll. Cardiol..

[18] Seals D.R., Moreau K.L., Gates P.E., Eskurza I. (2006). Modulatory influences on ageing of the vasculature in healthy humans. Exp. Gerontol..

[19] Clark M.G., Wallis M.G., Barrett E.J., Vincent M.A., Richards S.M., Clerk L.H., Rattigan S. (2003). Blood flow and muscle metabolism: A focus on insulin action. Am. J. Physiol. Endocrinol. Metab..

[20] Timmerman K.L., Dhanani S., Glynn E.L., Fry C.S., Drummond M.J., Jennings K., Rasmussen B.B., Volpi E. (2012). A moderate acute increase in physical activity enhances nutritive flow and the muscle protein anabolic response to mixed nutrient intake in older adults. Am. J. Clin. Nutr..

[21] Rodriguez A.J., Karim M.N., Srikanth V., Ebeling P.R., Scott D. (2017). Lower muscle tissue is associated with higher pulse wave velocity: A systematic review and meta-analysis of observational study data. Clin. Exp. Pharmacol. Physiol..

[22] Clark M.G. (2008). Impaired microvascular perfusion: A consequence of vascular dysfunction and a potential cause of insulin resistance in muscle. Am. J. Physiol. Endocrinol. Metab..

[23] Clark M.G., Rattigan S., Barrett E.J. (2006). Nutritive blood flow as an essential element supporting muscle anabolism. Curr. Opin. Clin. Nutr. Metab. Care.

[24] Durham W.J., Casperson S.L., Dillon E.L., Keske M.A., Paddon-Jones D., Sanford A.P., Hickner R.C., Grady J.J., Sheffield-Moore M. (2010). Age-related anabolic resistance after endurance-type exercise in healthy humans. FASEB J..

[25] Mitchell W.K., Phillips B.E., Williams J.P., Rankin D., Smith K., Lund J.N., Atherton P.J. (2013). Development of a new Sonovue contrast-enhanced ultrasound approach reveals temporal and age-related features of muscle microvascular responses to feeding. Physiol. Rep..

[26] Brightwell C.R., Markofski M.M., Moro T., Fry C.S., Porter C., Volpi E., Rasmussen B.B. (2019). Moderate-intensity aerobic exercise improves skeletal muscle quality in older adults. Transl. Sports Med..

[27] Verdijk L.B., Snijders T., Holloway T.M., Van Kranenburg J., Van Loon L.J. (2016). Resistance Training Increases Skeletal Muscle Capillarization in Healthy Older Men. Med. Sci. Sports Exerc..

[28] Rennie M.J. (2009). Anabolic resistance: The effects of aging, sexual dimorphism, and immobilization on human muscle protein turnover. Appl. Physiol. Nutr. Metab..

[29] Cuthbertson D., Smith K., Babraj J., Leese G., Waddell T., Atherton P., Wackerhage H., Taylor P.M., Rennie M.J. (2005). Anabolic signaling deficits underlie amino acid resistance of wasting, aging muscle. FASEB J..

[30] Wilkes E.A., Selby A.L., Atherton P.J., Patel R., Rankin D., Smith K., Rennie M.J. (2009). Blunting of insulin inhibition of proteolysis in legs of older subjects may contribute to age-related sarcopenia. Am. J. Clin. Nutr..

[31] Phillips B.E., Atherton P.J., Varadhan K., Limb M.C., Williams J.P., Smith K. (2016). Acute cocoa flavanol supplementation improves muscle macro- and microvascular but not anabolic responses to amino acids in older men. Appl. Physiol. Nutr. Metab..

[32] Fryar C.D., Ostchega Y., Hales C.M., Zhang G., Kruszon-Moran D. (2017). Hypertension Prevalence and Control among Adults: United States, 2015–2016. NCHS Data Brief.

[33] Barnouin Y., McPhee J.S., Butler-Browne G., Bosutti A., De Vito G., Jones D.A., Narici M., Behin A., Hogrel J.Y., Degens H. (2017). Coupling between skeletal muscle fiber size and capillarization is maintained during healthy aging. J. Cachexia Sarcopenia Muscle.

[34] Phillips B., Williams J., Atherton P., Smith K., Hildebrandt W., Rankin D., Greenhaff P., Macdonald I., Rennie M.J. (2012). Resistance exercise training improves age-related declines in leg vascular conductance and rejuvenates acute leg blood flow responses to feeding and exercise. J. Appl. Physiol..

[35] Barrera G., Bunout D., de la Maza M.P., Leiva L., Hirsch S. (2014). Carotid ultrasound examination as an aging and disability marker. Geriatr. Gerontol. Int..

[36] Chung J., Kim M., Jin Y., Kim Y., Hong J. (2018). Fitness as a determinant of arterial stiffness in healthy adult men: A cross-sectional study. J. Sports Med. Phys. Fit..

[37] den Ouden M.E., Schuurmans M.J., Arts I.E., Grobbee D.E., Bots M.L., van den Beld A.W., Lamberts S.W., van der Schouw Y.T. (2013). Atherosclerosis and physical functioning in older men, a longitudinal study. J. Nutr. Health Aging.

[38] El Khoudary S.R., Chen H.Y., Barinas-Mitchell E., McClure C., Selzer F., Karvonen-Gutierrez C., Jackson E.A., Ylitalo K.R., Sternfeld B. (2015). Simple physical performance measures and vascular health in late midlife women: The Study of Women’s Health across the nation. Int. J. Cardiol..

[39] Fahs C.A., Thiebaud R.S., Rossow L.M., Loenneke J.P., Bemben D.A., Bemben M.G. (2018). Relationships between central arterial stiffness, lean body mass, and absolute and relative strength in young and older men and women. Clin. Physiol. Funct. Imaging.

[40] Gonzales J.U., Wiberg M., Defferari E., Proctor D.N. (2015). Arterial stiffness is higher in older adults with increased perceived fatigue and fatigability during walking. Exp. Gerontol..

[41] Heffernan K.S., Chale A., Hau C., Cloutier G.J., Phillips E.M., Warner P., Nickerson H., Reid K.F., Kuvin J.T., Fielding R.A. (2012). Systemic vascular function is associated with muscular power in older adults. J. Aging Res..

[42] Im I.J., Choi H.J., Jeong S.M., Kim H.J., Son J.S., Oh H.J. (2017). The association between muscle mass deficits and arterial stiffness in middle-aged men. Nutr. Metab. Cardiovasc. Dis..

[43] Kohara K., Okada Y., Ochi M., Ohara M., Nagai T., Tabara Y., Igase M. (2017). Muscle mass decline, arterial stiffness, white matter hyperintensity, and cognitive impairment: Japan Shimanami Health Promoting Program study. J. Cachexia Sarcopenia Muscle.

[44] Lee S.W., Youm Y., Kim C.O., Lee W.J., Choi W., Chu S.H., Park Y.R., Kim H.C. (2014). Association between skeletal muscle mass and radial augmentation index in an elderly Korean population. Arch. Gerontol. Geriatr..

[45] Mitchell W.K., Williams J., Atherton P., Larvin M., Lund J., Narici M. (2012). Sarcopenia, dynapenia, and the impact of advancing age on human skeletal muscle size and strength; a quantitative review. Front. Physiol..

[46] Ochi M., Kohara K., Tabara Y., Kido T., Uetani E., Ochi N., Igase M., Miki T. (2010). Arterial stiffness is associated with low thigh muscle mass in middle-aged to elderly men. Atherosclerosis.

[47] Prior S.J., Ryan A.S., Blumenthal J.B., Watson J.M., Katzel L.I., Goldberg A.P. (2016). Sarcopenia Is Associated With Lower Skeletal Muscle Capillarization and Exercise Capacity in Older Adults. J. Gerontol. Ser. A Biol. Sci. Med Sci..

[48] Sanada K., Miyachi M., Tanimoto M., Yamamoto K., Murakami H., Okumura S., Gando Y., Suzuki K., Tabata I., Higuchi M. (2010). A cross-sectional study of sarcopenia in Japanese men and women: Reference values and association with cardiovascular risk factors. Eur. J. Appl. Physiol..

[49] Shiotsu Y., Watanabe Y., Tujii S., Yanagita M. (2018). Effect of exercise order of combined aerobic and resistance training on arterial stiffness in older men. Exp. Gerontol..

[50] Suwa M., Imoto T., Kida A., Yokochi T., Iwase M., Kozawa K. (2018). Association of body flexibility and carotid atherosclerosis in Japanese middle-aged men: A cross-sectional study. BMJ Open.

[51] Yamamoto K., Kawano H., Gando Y., Iemitsu M., Murakami H., Sanada K., Tanimoto M., Ohmori Y., Higuchi M., Tabata I. (2009). Poor trunk flexibility is associated with arterial stiffening. Am. J. Physiol. Heart Circ. Physiol..

[52] Yoshizawa M., Maeda S., Miyaki A., Misono M., Saito Y., Tanabe K., Kuno S., Ajisaka R. (2009). Effect of 12 weeks of moderate-intensity resistance training on arterial stiffness: A randomised controlled trial in women aged 32–59 years. Br. J. Sports Med..

[53] Yoo J.I., Kim M.J., Na J.B., Chun Y.H., Park Y.J., Park Y., Hah Y.S., Ha Y.C., Park K.S. (2018). Relationship between endothelial function and skeletal muscle strength in community dwelling elderly women. J. Cachexia Sarcopenia Muscle.

[54] Zhang L., Guo Q., Feng B.L., Wang C.Y., Han P.P., Hu J., Sun X.D., Zeng W.F., Zheng Z.X., Li H.S. (2019). A Cross-Sectional Study of the Association between Arterial Stiffness and Sarcopenia in Chinese Community-Dwelling Elderly Using the Asian Working Group for Sarcopenia Criteria. J. Nutr. Health Aging.

[55] Dipla K., Triantafyllou A., Koletsos N., Papadopoulos S., Sachpekidis V., Vrabas I.S., Gkaliagkousi E., Zafeiridis A., Douma S. (2017). Impaired Muscle Oxygenation and Elevated Exercise Blood Pressure in Hypertensive Patients: Links With Vascular Stiffness. Hypertension.

[56] Gueugneau M., Coudy-Gandilhon C., Meunier B., Combaret L., Taillandier D., Polge C., Attaix D., Roche F., Feasson L., Barthelemy J.C. (2016). Lower skeletal muscle capillarization in hypertensive elderly men. Exp. Gerontol..

[57] Lima-Junior D., Farah B.Q., Germano-Soares A.H., Andrade-Lima A., Silva G.O., Rodrigues S.L.C., Ritti-Dias R. (2018). Association between handgrip strength and vascular function in patients with hypertension. Clin. Exp. Hypertens..

[58] Sampaio R.A., Sewo Sampaio P.Y., Yamada M., Yukutake T., Uchida M.C., Tsuboyama T., Arai H. (2014). Arterial stiffness is associated with low skeletal muscle mass in Japanese community-dwelling older adults. Geriatr. Gerontol. Int..

[59] Shimizu Y., Sato S., Koyamatsu J., Yamanashi H., Nagayoshi M., Kadota K., Kawashiri S.Y., Inoue K., Nagata Y., Maeda T. (2017). Handgrip strength and subclinical carotid atherosclerosis in relation to platelet levels among hypertensive elderly Japanese. Oncotarget.

[60] Wong A., Figueroa A., Son W.M., Chernykh O., Park S.Y. (2018). The effects of stair climbing on arterial stiffness, blood pressure, and leg strength in postmenopausal women with stage 2 hypertension. Menopause.

[61] Groen B.B., Hamer H.M., Snijders T., van Kranenburg J., Frijns D., Vink H., van Loon L.J. (2014). Skeletal muscle capillary density and microvascular function are compromised with aging and type 2 diabetes. J. Appl. Physiol..

[62] Wust R.C., Gibbings S.L., Degens H. (2009). Fiber capillary supply related to fiber size and oxidative capacity in human and rat skeletal muscle. Adv. Exp. Med. Biol..

[63] Bodur H., Yilmaz O., Keskin D. (2006). Hand disability and related variables in patients with rheumatoid arthritis. Rheumatol. Int..

[64] Escalante A., Haas R.W., del Rincon I. (2004). Measurement of global functional performance in patients with rheumatoid arthritis using rheumatology function tests. Arthritis Res. Ther..

[65] Leong D.P., Teo K.K., Rangarajan S., Lopez-Jaramillo P., Avezum A., Orlandini A., Seron P., Ahmed S.H., Rosengren A., Kelishadi R. (2015). Prognostic value of grip strength: Findings from the Prospective Urban Rural Epidemiology (PURE) study. Lancet.

[66] Abellan van Kan G., Rolland Y., Andrieu S., Bauer J., Beauchet O., Bonnefoy M., Cesari M., Donini L.M., Gillette Guyonnet S., Inzitari M. (2009). Gait speed at usual pace as a predictor of adverse outcomes in community-dwelling older people an International Academy on Nutrition and Aging (IANA) Task Force. J. Nutr. Health Aging.

[67] Afilalo J., Eisenberg M.J., Morin J.F., Bergman H., Monette J., Noiseux N., Perrault L.P., Alexander K.P., Langlois Y., Dendukuri N. (2010). Gait speed as an incremental predictor of mortality and major morbidity in elderly patients undergoing cardiac surgery. J. Am. Coll. Cardiol..

[68] Blain H., Carriere I., Sourial N., Berard C., Favier F., Colvez A., Bergman H. (2010). Balance and walking speed predict subsequent 8-year mortality independently of current and intermediate events in well-functioning women aged 75 years and older. J. Nutr. Health Aging.

[69] Ries J.D., Echternach J.L., Nof L., Gagnon Blodgett M. (2009). Test-retest reliability and minimal detectable change scores for the timed “up & go” test, the six-minute walk test, and gait speed in people with Alzheimer disease. Phys. Ther..

[70] Studenski S., Perera S., Patel K., Rosano C., Faulkner K., Inzitari M., Brach J., Chandler J., Cawthon P., Connor E.B. (2011). Gait speed and survival in older adults. JAMA.

[71] Rodriguez A.J., Scott D., Ebeling P.R. (2019). Exploring the Links Between Common Diseases of Ageing—Osteoporosis, Sarcopenia and Vascular Calcification. Clin. Rev. Bone Miner. Metab..

[72] Andrade J., Er L., Ignaszewski A., Levin A. (2008). Exploration of association of 1,25-OH2D3 with augmentation index, a composite measure of arterial stiffness. Clin. J. Am. Soc. Nephrol..

[73] Khan B., Nowson C.A., Daly R.M., English D.R., Hodge A.M., Giles G.G., Ebeling P.R. (2015). Higher Dietary Calcium Intakes Are Associated With Reduced Risks of Fractures, Cardiovascular Events, and Mortality: A Prospective Cohort Study of Older Men and Women. J. Bone Miner. Res..

[74] Rodriguez A.J., Scott D., Srikanth V., Ebeling P. (2016). Effect of vitamin D supplementation on measures of arterial stiffness: A systematic review and meta-analysis of randomized controlled trials. Clin. Endocrinol..

[75] Anderson J.J., Kruszka B., Delaney J.A., He K., Burke G.L., Alonso A., Bild D.E., Budoff M., Michos E.D. (2016). Calcium Intake From Diet and Supplements and the Risk of Coronary Artery Calcification and its Progression Among Older Adults: 10-Year Follow-up of the Multi-Ethnic Study of Atherosclerosis (MESA). J. Am. Heart Assoc..

[76] Kim J.H., Yoon J.W., Kim K.W., Lee E.J., Lee W., Cho S.H., Shin C.S. (2012). Increased dietary calcium intake is not associated with coronary artery calcification. Int. J. Cardiol..

[77] Nocon M., Hiemann T., Muller-Riemenschneider F., Thalau F., Roll S., Willich S.N. (2008). Association of physical activity with all-cause and cardiovascular mortality: A systematic review and meta-analysis. Eur. J. Cardiovasc. Prev. Rehabil..

[78] Sofi F., Capalbo A., Cesari F., Abbate R., Gensini G.F. (2008). Physical activity during leisure time and primary prevention of coronary heart disease: An updated meta-analysis of cohort studies. Eur. J. Cardiovasc. Prev. Rehabil..

[79] Melo X., Santa-Clara H., Santos D.A., Pimenta N.M., Minderico C.S., Fernhall B., Sardinha L.B. (2015). Independent Association of Muscular Strength and Carotid Intima-Media Thickness in Children. Int. J. Sports Med..

[80] Badrov M.B., Freeman S.R., Zokvic M.A., Millar P.J., McGowan C.L. (2016). Isometric exercise training lowers resting blood pressure and improves local brachial artery flow-mediated dilation equally in men and women. Eur. J. Appl. Physiol..

